# Investigating the predictive value of vascular endothelial growth factor in the evaluation of treatment efficacy and prognosis for patients with non-surgical esophageal squamous cell carcinoma

**DOI:** 10.3389/fonc.2022.843250

**Published:** 2022-10-26

**Authors:** Ze Kong, Fei Sun, Qinghong Meng, Mengyun Zhou, Jingping Yu, Lijun Hu

**Affiliations:** ^1^ Department of Radiation Oncology, The Affiliated Changzhou No. 2 People’s Hospital of Nanjing Medical University, Changzhou, Jiangsu, China; ^2^ Department of Radiation Oncology, ShuGuang Hospital Affiliated to Shanghai University of Chinese Traditional Medicine, Shanghai, China

**Keywords:** esophageal cancer, squamous cell carcinoma, vascular endothelial growth factor, treatment efficacy, prognosis, model

## Abstract

In this study, we aim to investigate the predictive value of serum vascular endothelial growth factor (VEGF) in evaluating treatment efficacy and long-term prognosis for patients with non-surgical esophageal squamous cell carcinoma (ESCC). The patients diagnosed with ESCC by histopathology who didn’t receive surgical treatment were retrospectively analyzed. Through follow-up and prognostic analysis, we explored the value of serum VEGF changes before, during, and after radiotherapy for predicting treatment efficacy, and identified important indicators to construct the predictive model. Eighty-four patients were enrolled in this study, and the objective response rate (ORR) after treatment was 75.0%. The serum VEGF before, during and after radiotherapy were 108.2 ± 38.4, 98.6 ± 20.3 and 96.9 ± 20.0pg/ml, respectively. Staging and serum VEGF during radiotherapy were the independent factors affecting the treatment efficacy of non-surgical ESCC patients (OR=0.182 and 0.959, P<0.05). The median overall survival (OS) and progression-free survival (PFS) were 24.4 and 15.8 months. The 3-year, 5-year, 10-year OS rates and PFS rates were 35.7%, 26.2%, 14.4%, and 26.2%, 22.6%, 12.3%, respectively. By performing COX regression analysis, we found that the TNM stage, changes of VEGF after radiotherapy (∆VEGF2), and endoscopic histopathological response were the independent prognostic factors for OS and PFS (P<0.05). The R^2^ of the constructed prediction model was 0.328 and 0.362, and the C-index was 0.697 and 0.708, respectively. The follow-up time-dependent AUC showed that the predicted AUC was stable and greater than 0.7 as the follow-up time increased. For patients with non-surgical ESCC, those with low VEGF levels during radiotherapy had better treatment efficacy, and those with significant VEGF reduction after radiotherapy had a better prognosis. In summary, our results demonstrate that it is feasible to construct a model to evaluate and predict the efficacy and prognosis of patients with non-surgical ESCC based on serum VEGF measurement.

## Introduction

Esophageal cancer is one of the most common malignant tumors of the digestive system, with strong invasiveness and poor prognosis. According to the statistics, in 2020, there are 604,000 new cases of esophageal cancer and 544,000 deaths worldwide, ranked 7th and 6th among all malignant tumors; moreover, one in 18 cancer-related deaths is esophageal cancer (1). Among the new esophageal cancer cases, 85% are esophageal squamous cell carcinoma (ESCC) (2). Due to the insidious onset of the disease, more than 50% of patients with ESCC are already at the locally advanced stage at the time of diagnosis and unable to receive surgery (3). So far, the treatment strategies for locally advanced esophageal cancer are divided into surgical and non-surgical types according to whether radical resection can be performed (4). For patients with non-surgical ESCC, concurrent radiochemotherapy is the main radical treatment (4, 5). Although the objective response rate through radical treatment can reach 53.3-98.3% (6), the failure rate in local areas is still above 50%, and the 5-year survival rate is only 15-37% (7, 8). Therefore, to improve the local control rate of non-surgical ESCC patients and reduce local recurrence or persistence are the keys to improving the survival and prognosis of such patients.

Tumor angiogenesis and lymphangiogenesis are key biological behaviors of malignant tumors. The genes that promote angiogenesis are induced when the tumor volume increases and the central area is hypoxic. Vascular endothelial growth factor (VEGF) is a member of the platelet-derived growth factor family; it is a highly specific mitogen in vascular endothelial cells, and is the most effective pro-angiogenic factor (9). The VEGF/VEGFR interaction can trigger a variety of signaling pathways, such as extracellular regulated protein kinase 1/2 (ERK1/2) and phosphatidylinositol 3-kinase/protein kinase B (PI3K/AKT) pathways, leading to tumor cell proliferation, migration and survival (10, 11). It has been shown that the serum VEGF in patients with various solid tumors (such as lung cancer (12), breast cancer (13), gastric cancer (14), etc.) are significantly elevated. Similarly, VEGF also plays a vital role in the progression of esophageal cancer. Shimada et al. (15) found that the serum VEGF level in patients with esophageal squamous cell carcinoma (ESCC) was significantly increased, and that high serum VEGF levels were closely related to increased tumor staging, poor radiotherapy and chemotherapy effects, and poor prognosis. However, some studies found that VEGF levels in non-surgical ESCC patients were not significantly correlated with overall survival (OS) or progression-free survival (PFS) before treatment (16), but the trend of VEGF changes after treatment is the prognostic factor for non-surgical ESCC patients. Therefore, the value of serum VEGF changes in predicting the efficacy and prognosis of non-surgical ESCC is unclear. In this retrospective study, we aimed to elucidate the importance of dynamic monitoring of serum VEGF in predicting the efficacy and prognosis of non-surgical ESCC patients.

## Materials and methods

### Clinical information

A retrospective analysis was performed on patients with ESCC admitted to the Department of Radiotherapy in our hospital from May 2008 to December 2014 and diagnosed *via* histopathology. Enrollment criteria: 1) Age ≥18 years, 2) KPS score ≥80, 3) Not eligible for surgery or refusal of surgery for other reasons, 4) Received radical radiotherapy: the radiotherapy dose was ≥50Gy, and no antiangiogenic therapy was performed during the same period, 5) No previous anti-tumor treatment, 6) Liquid diet at least, 7) Complete clinical and follow-up data. Exclusion criteria: 1) Gastroscopy or esophageal barium dialysis suggested deep ulcers or burrs on the esophageal wall, 2) Active bleeding or severe coagulation dysfunction, 3) Esophageal lesions > 10 cm or extending to stomach for more than 2 cm, 4) Combined with the second primary tumor, 5) Severe heart and lung diseases. The patient’s age, sex, history of hypertension and diabetes, location and type of esophageal cancer, staging (according to the 2010 China Clinical staging Criteria for Nonoperative Treatment of Esophageal Carcinoma (17)), tumor markers (CEA, SCCA), serum VEGF and changes, treatment method and follow-up information during treatment were recorded. All patients signed an informed consent form before treatment. This study was approved by the ethics committee of our hospital.

### Treatment method

#### Radiotherapy

The 6MV X-ray three-dimensional conformal radiotherapy or intensity-modulated radiotherapy was used, and the radiotherapy plan was designed by CT-SIM. Gross tumor volume (GTV): GTV included esophageal lesions and metastatic lymph nodes; it was comprehensively assessed based on localized CT images combined with gastroscopy, esophageal barium perfusion, and cervical-thoracic enhanced CT. Clinical target volume (CTV): CTV was expanded based on GTV; esophageal lesions were expanded for 0.5~1 cm in the front and back, left and right directions, and 3 cm in the up and down direction (along the esophagus, appropriate adjustments were made when encountering anatomical barriers); the metastatic lymph nodes were uniformly expanded for 0.5~1 cm. Planning target volume (PTV): PTV was uniformly expanded for 0.5 cm based on CTV. The prescribed dose of PTV was 50-66 Gy, 1.8-2.0 Gy/time, once/day, 5 days/week, and routinely divided. The average lung dose was ≤13Gy; the two lungs V5 ≤ 60%, V20 ≤ 28%; the heart V40 ≤ 45%; the maximum dose for the spinal cord was ≤45Gy.

#### Chemotherapy

Day 1: paclitaxel liposome 135 mg/m^2^ intravenous infusion; Day 1-4: cisplatin 20 mg/m^2^ intravenous infusion. 21 days was one treatment cycle. 2 cycles of chemotherapy were performed together with radiotherapy, and 2 cycles were conducted after radiotherapy as consolidation chemotherapy.

### Determination of serum VEGF levels

2 ml of peripheral venous blood was drawn at three time points: before radiotherapy, during radiotherapy (1 month after radiotherapy started), and within 1 week after radiotherapy. The blood sample was centrifuged at 4°C, 3000 r/min for 10 min. After centrifugation, the serum was stored at -80°C for the following test. The VEGF concentration in the serum samples was determined by ELISA using VEGF ELISA kit (NeoBioscience, Shenzhen, China) according to the manufacturer’s instructions. Before running the assay, the samples were thawed at 4°C overnight and then 30-fold diluted with diluent. The changes in serum VEGF levels during and after radiotherapy were calculated. The calculation formula was as follows: ∆VEGF1=(serum VEGF during radiotherapy- serum VEGF before radiotherapy)/VEGF before radiotherapy, ∆VEGF2=(serum VEGF after radiotherapy- serum VEGF before radiotherapy)/VEGF Before radiotherapy.

### Pathological evaluation of endoscope

All patients underwent electronic gastroscopy and pathological biopsy on the 4th week after the start of radiotherapy. According to the histopathological characteristics under the microscope, the treatment response was classified into 3 levels: mild, moderate, and obvious response (18): mild response: tumor cells showed mild degenerative changes, reduced mitosis index, small degrees of inflammatory cell infiltration and vascular proliferation; moderate response: most of the tumor cells disappeared, the remaining tumors showed degenerative changes and were mostly wrapped by granulation tissue, more inflammatory cells infiltrated; obvious response: tumor cells disappeared completely, fibrous tissue proliferated in the tumor bed, blood vessels decreased, chronic inflammatory cell infiltrated, scar formed.

### Short-term efficacy evaluation, follow-up, and side effects

According to the RECIST 1.1 criteria, the treatment efficacy was divided into complete response (CR), partial response (PR), stable disease (SD) and disease progression (PD). The clinical response includes CR and PR. Follow-up was conducted every 3 months within 2 years after treatment, every 6 months after 2 years, and every year after 5 years. The follow-up ended on December 31, 2020, with a median follow-up time of 27 months (6 to 150 months). The overall survival (OS) and progression-free survival (PFS) were used as the prognosis evaluation parameters. OS is defined as the time from the start of treatment until death from any cause or the last follow-up date. PFS was defined as the time between the start of treatment and the first event of local failure, metastatic recurrence, progression, or death. Acute radiotherapy adverse reactions were evaluated according to Radiation therapy oncology group (RTOG) acute radiation injury grading criteria. Chemotherapy adverse reactions were evaluated according to Common Terminology Criteria for Adverse Events version 4.0.

### Statistical methods

Statistical analysis was performed using R (version 3.4.3, http://www.R-project.org). Normally distributed measurement data were expressed as mean ± standard deviation, non-normally distributed measurement data were expressed as median P50 (P25, P75), and count data/categorical variables were expressed as number or percentage. Unpaired Student-t test or Mann-Whitney u nonparametric test was used for comparing continuous variables between two groups. One-way analysis of variance (normally distributed continuous variables) and Kruskal-Wallis test (skew continuous variables) were used for comparing three groups or more. The Chi-square test or Fisher’s exact test was used to analyze the differences between categorical variables. The correlation between the two variables was analyzed by Pearson or Spearman correlation analysis. The single-index receiver operating characteristic (ROC) curve was plotted to obtain the sensitivity, specificity, area under curve (AUC), and 95% confidence interval (CI) of each index for predicting CR after treatment. DeLong and other methods were used to compare the statistical differences of AUC. Logistic regression analysis was used for multivariate analysis of two categorical variables. Survival analysis was performed using Kaplan-Meier method and log-rank method. COX univariate and multivariate regression analyses were used to build the predictive model (variable introduction standard P<0.05). The best model parameters were selected according to the minimum Akaike’s information criterion (AIC). The consistency index (C index), which evaluated the quality of COX regression model, was calculated, and the predictive nomogram, and follow-up time-dependent AUC curve were plotted (19). P<0.05 was considered statistically significant.

## Results

### Clinical data

A total of 84 patients with ESCC met the enrollment criteria, with an average age of 66.3 ± 10.1 years (40-87 years). 71.4% (60/84) of the patients were male, and 73.8% (62/84) received concurrent radiotherapy and chemotherapy. The serum VEGF levels before, during and after radiotherapy were 108.2 ± 38.4 pg/ml, 98.6 ± 20.3 pg/ml and 96.9 ± 20.0 pg/ml, respectively. The clinical data and pathological characteristics are shown in **Table 1**.

**Table 1:** Clinical data of study subjects.

Parameters	Study subjects (n=84)
**Male**	60 (71.4%)
**Age ≥65 years**	50 (59.5%)
**Hypertension**	30 (35.7%)
**Diabetes**	12 (14.3%)
**CEA (ng/ml)**	2.17 ± 1.69
**SCCA (ng/ml)**	1.95 ± 1.80
**Diet before treatment**	
Liquid food	8 (9.5%)
Semi-liquid food	12 (14.3%)
Soft food	64 (76.2%)
**Tumor location**	
Neck, upper chest	22 (26.2%)
Mid chest	37 (44.1%)
Lower chest	25 (29.7%)
**Tumor classification**	
Medullary	79 (94.1%)
Ulcer, constricted	5 (5.9%)
**T staging**	
T_1-2_	14 (16.7%)
T_3_	54 (64.2%)
T_4_	16 (19.1%)
**N staging**	
N_0_	15 (17.9%)
N_1-2_	69 (82.1%)
**Staging**	
Stage I	6 (7.1%)
Stage II	62 (73.8%)
Stage III	16 (19.1%)
**Treatment strategy**	
Concurrent radiochemotherapy	62 (73.8%)
Radiotherapy alone	22 (26.2%)

CEA, carcinoembryonic antigen, SCCA, squamous cell carcinoma antigen.

### Changes of serum VEGF in non-surgical ESCC patients before, during and after radiotherapy

All patients tolerated and completed the planned treatment regimen. After receiving concurrent chemoradiotherapy or radiotherapy alone, 75.0% (63/84) of the non-surgical ESCC patients were in clinical response state (CR+PR), and 25.0% (21/84) did not respond to treatment (SD+PD). The objective response rate (ORR) was 75.0%. The esophageal toxicity rates for grade 1, 2, 3 were 22.6%, 69.0% and 8.3%, respectively. The lung toxicity rates for grade 1, 2 were 61.9%, 38.1%, respectively. The blood toxicity rates for grade 1, 2, 3, and 4 were 20.2%, 25.0%, 28.6%, and 8.3%, respectively.

**Figure 1:**
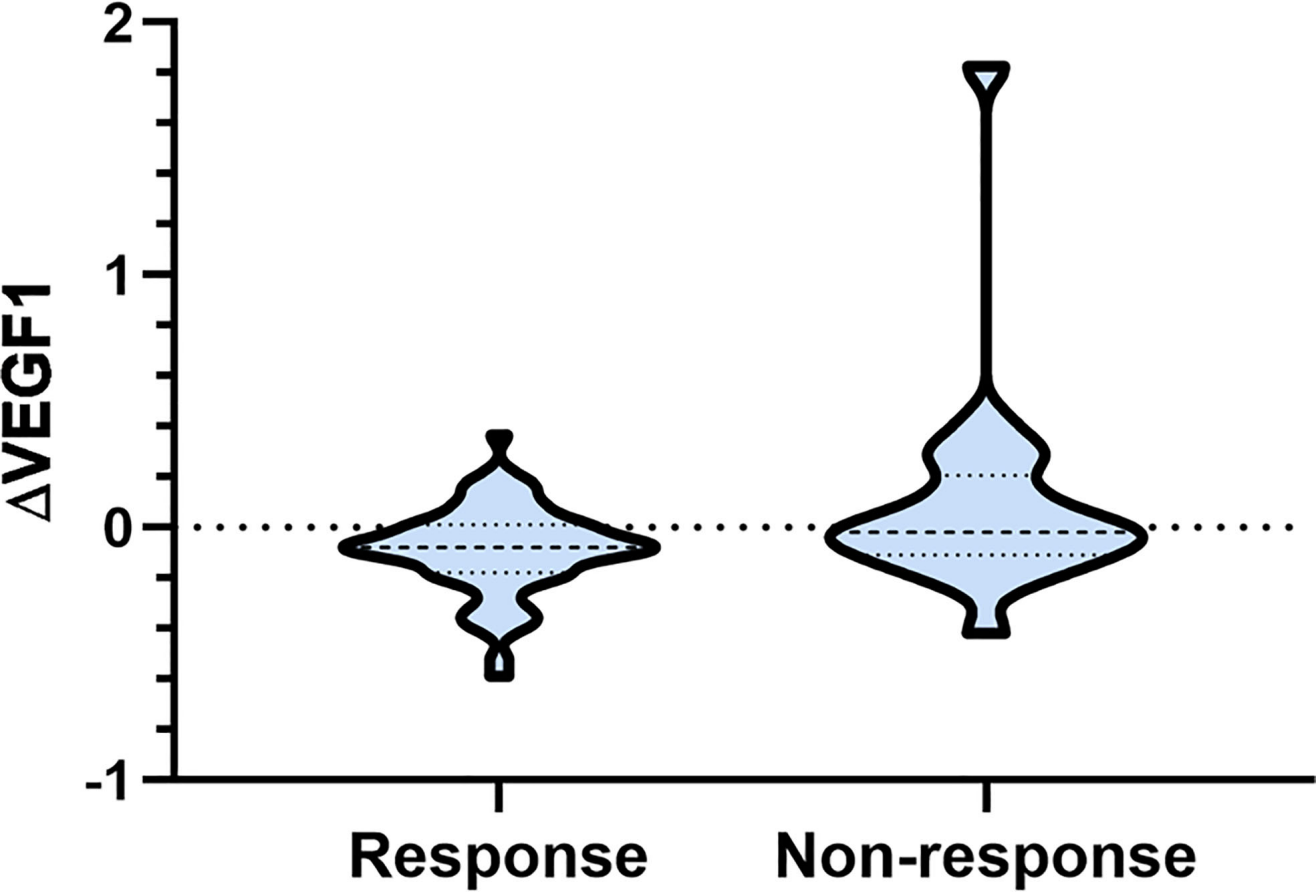
Comparison of serum ∆VEGF1 in different response groups after radiotherapy.

As shown in **Table 2**, there was no significant difference in serum VEGF before and after radiotherapy, and the ∆VEGF2 levels between the response and non-response groups were similar (P>0.05); the serum VEGF level during radiotherapy in the response group was significantly lower than that in the non-response group (P<0.001), and the difference in ∆VEGF1 was more significant (P=0.042, **Figure 1**).

**Table 2:** Changes of blood VEGF before and after radiotherapy in response group and non-response group.

Parameter	Response group (n=63)	Non-response group (n=21)	*t/Z* value	*P* value
VEGF before RT (pg/ml)	108.6 ± 34.6	107.1 ± 25.1	-0.174	0.862
VEGF during RT (pg/ml)	94.3 ± 16.2	111.6 ± 25.8	3.619	<0.001^*^
VEGF after RT (pg/ml)	95.3 ± 18.6	101.9 ± 23.6	1.324	0.189
∆VEGF1 (%)	-7.9 (-17.3, 0.6)	-2.2 (-10.6, 14.6)	-2.035	0.042^*#^
∆VEGF2 (%)	-9.5 (-15.7, -2.4)	-8.0 (-21.0, 12.6)	-0.801	0.435^#^

RT, radiotherapy; ∆VEGF1=(serum VEGF _during radiotherapy_- serum VEGF _before radiotherapy_)/VEGF _before radiotherapy_, ∆VEGF2=(serum VEGF _after radiotherapy_- serum VEGF _before radiotherapy_)/VEGF _Before radiotherapy_. ^*^Difference is statistically significant (P<0.05), ^#^Mann Whitney u test.

**Figure 2:**
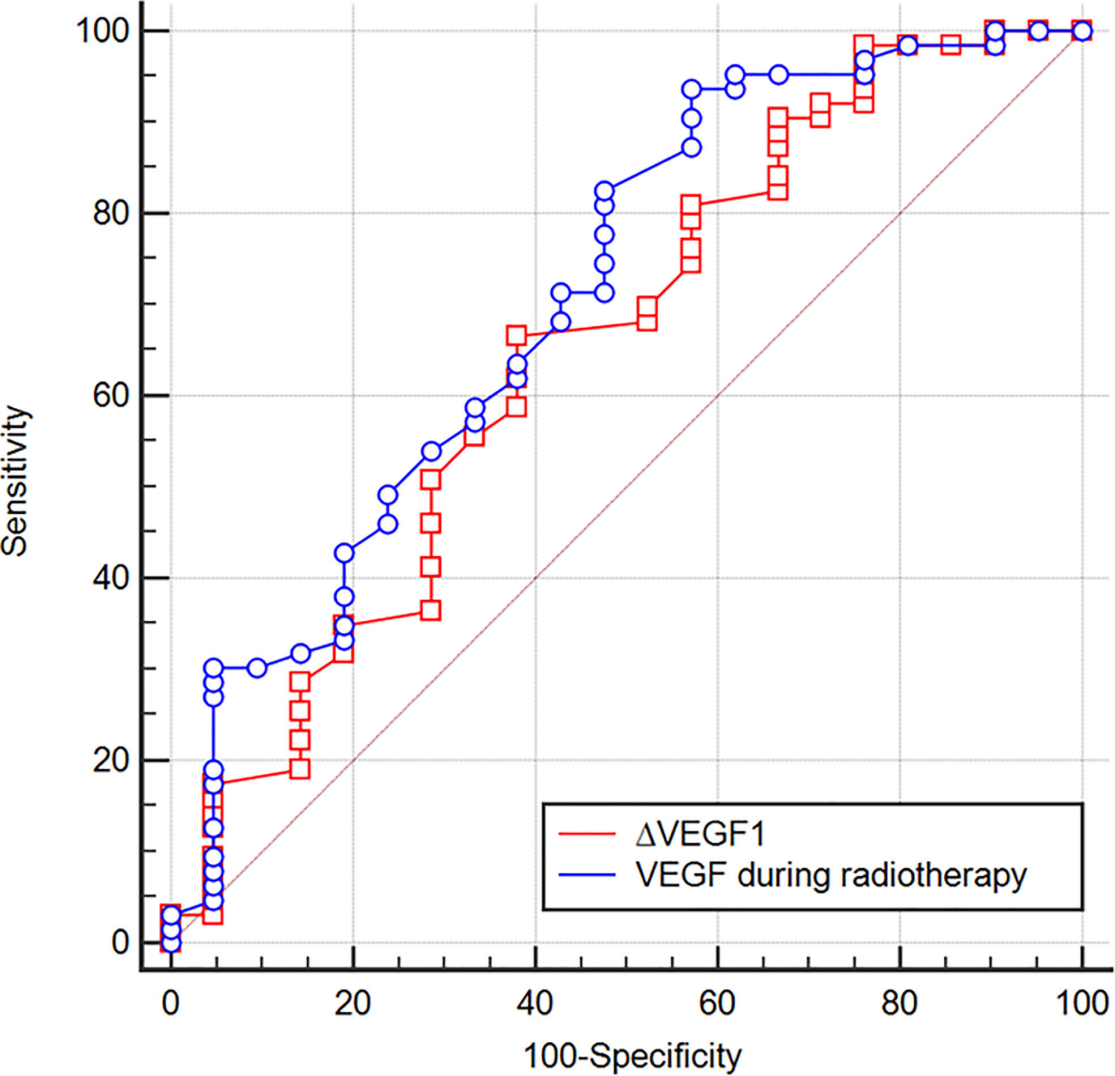
ROC curves of using VEGF during radiotherapy and ∆VEGF1 to predict the treatment efficacy of ESCC. When VEGF = 115 pg/ml during radiotherapy was used as the cut-off value to predict the short-term efficacy, the AUC was 0.710, the sensitivity was 93.7%, the specificity was 42.9%, z=2.997, P=0.003; when ∆VEGF1 = -4% was used as the cut-off value to predict the short-term efficacy, the AUC was 0.651, the sensitivity was 66.7%, the specificity was 61.9%, z=2.042, P=0.041.

The ROC curve was plotted to analyze whether the level of VEGF during radiotherapy and the change (∆VEGF1) during radiotherapy can be used to predict the short-term efficacy of esophageal cancer treatment. We found that, when using VEGF=115 pg/ml as the cut-off value to predict short-term efficacy, the AUC was 0.710, the sensitivity was 93.7%, the specificity was 42.9%, z=2.997, and P=0.003; when ∆VEGF1=-4% was used as the cut-off value to predict the short-term efficacy, the AUC was 0.651, the sensitivity was 66.7%, the specificity was 61.9%, z=2.042, and P=0.041. The AUC of using VEGF during radiotherapy to predict short-term efficacy was slightly larger than ∆VEGF1, but the difference was not statistically significant (Z=0.759, P=0.448, **Figure 2**). Through univariate logistic regression analysis, we found that the patients’ stage, treatment method, VEGF level during radiotherapy, and ∆VEGF1 were the influencing factors of short-term efficacy. In addition, multivariate logistic regression analysis (Y: response is 1, non-response is 0) showed that stage and VEGF level during radiotherapy were the independent influencing factors for short-term efficacy (**Table 3**), suggesting that patients with early stage and low VEGF level during radiotherapy were more likely to show better treatment efficacy.

**Table 3:** Multivariate logistic regression analysis for factors affecting efficacy.

Parameter	Regression coefficients	*OR*	95% *CI*	*P*
Staging	-1.706	0.182	0.048~0.681	0.011^*^
Treatment method	0.991	2.693	0.722~10.039	0.140
VEGF during RT	-0.042	0.959	0.925~0.994	0.024^*^
∆VEGF1	-2.847	0.058	0.002~1.918	0.111

^*^: the difference is statistically significant (P<0.05).

### The effect of serum VEGF levels during and after radiotherapy on survival analysis

**Figure 3:**
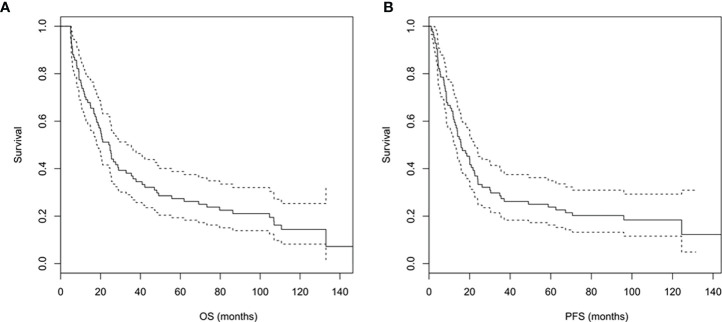
**(A, B)** Overall survival **(A)** and progression-free survival **(B)** curves of the study population.

The median OS of the study population was 24.4 (95% CI: 18.1 to 35.5) months, and the 1-year, 3-year, 5-year, and 10-year OS rates were 70.2%, 35.7%, 26.2%, and 14.4%, respectively (**Figure 3A**). The median PFS was 15.8 (95% CI: 12.5 to 22.6) months, and the 1-year, 3-year, 5-year, and 10-year PFS rates were 60.7%, 26.2%, 22.6%, and 12.3%, respectively (**Figure 3B**).

**Figure 4:**
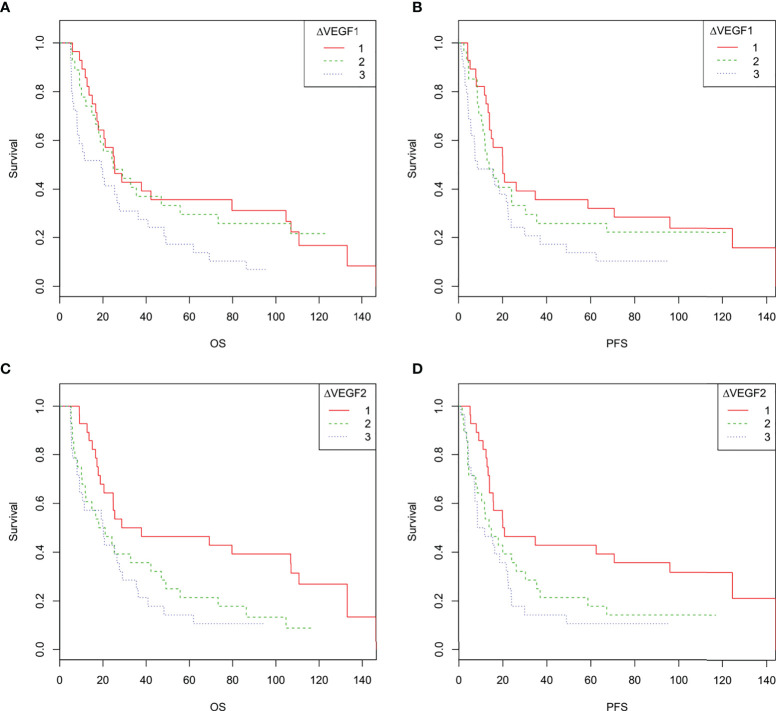
**(A–D)**. Overall survival and progression-free survival curves of patients with different changes in VEGF during and after radiotherapy. 1 indicates decreased VEGF, 2 indicates stable VEGF, 3 indicates increased VEGF. **(A, C)**: The overall survival curve of ESCC patients with different changes in VEGF during radiotherapy **(A)** and after radiotherapy **(C, D)**, the progression-free survival curves of ESCC patients with different changes in VEGF during radiotherapy **(B)** and after radiotherapy **(D)**.

According to the tertiles of serum VEGF changes during and after radiotherapy (∆VEGF1 and ∆VEGF2), all patients were divided into 3 groups, which were defined as the VEGF reduction group during radiotherapy [-20.2% (-34.0%, -15.8%)], stable group during radiotherapy [-7.0% (-8.7%, -4.4%)], elevated group during radiotherapy [9.9% (2.9%, 17.9%)], VEGF reduction group after radiotherapy [-21.7% (-28.7%, -17.0%))], stable group after radiotherapy [-9.4% (-11.5%, -5.7%)], and elevated group after radiotherapy [7.4% (1.1%, 28.3%)]. As shown in **Table 4** and **Figure 4**, the univariate regression analysis found that the change of VEGF (∆VEGF1) during radiotherapy was an influencing factor for the OS, but it had no significant effect on the PFS; while the change of VEGF (∆VEGF2) after radiotherapy had a significant effect on both OS and PFS.

**Table 4:** Survival analysis of VEGF reduction group, stable group and elevated group during/after radiotherapy.

Variable		Reduction group	Stable group	Elevated group	χ^2^	*P*
**OS (months)**	**During RT**	25.3 (17.8-107.0)	24.8 (18.1-73.3)	19.2 (8.2-36.3)	6.210	0.045^*^
	**After RT**	33.3 (20.5-115.5)	19.5 (11.8-49.1)	20.0 (9.2-35.5)	8.230	0.016^*^
**PFS (months)**	**During RT**	20.1 (14.1-96.0)	13.9 (11.1-35.5)	8.6 (5.7-22.6)	4.543	0.103
	**After RT**	20.5 (14.1-96.5)	14.4 (8.5-35.5)	10.2 (7.4-22.6)	7.140	0.028^*^

Data are expressed as median (95% CI). OS: overall survival, PFS: progression-free survival. *indicates that the difference is statistically significant (P<0.05).

### Construction of a model for predicting the prognosis of non-surgical ESCC

**Figure 5:**
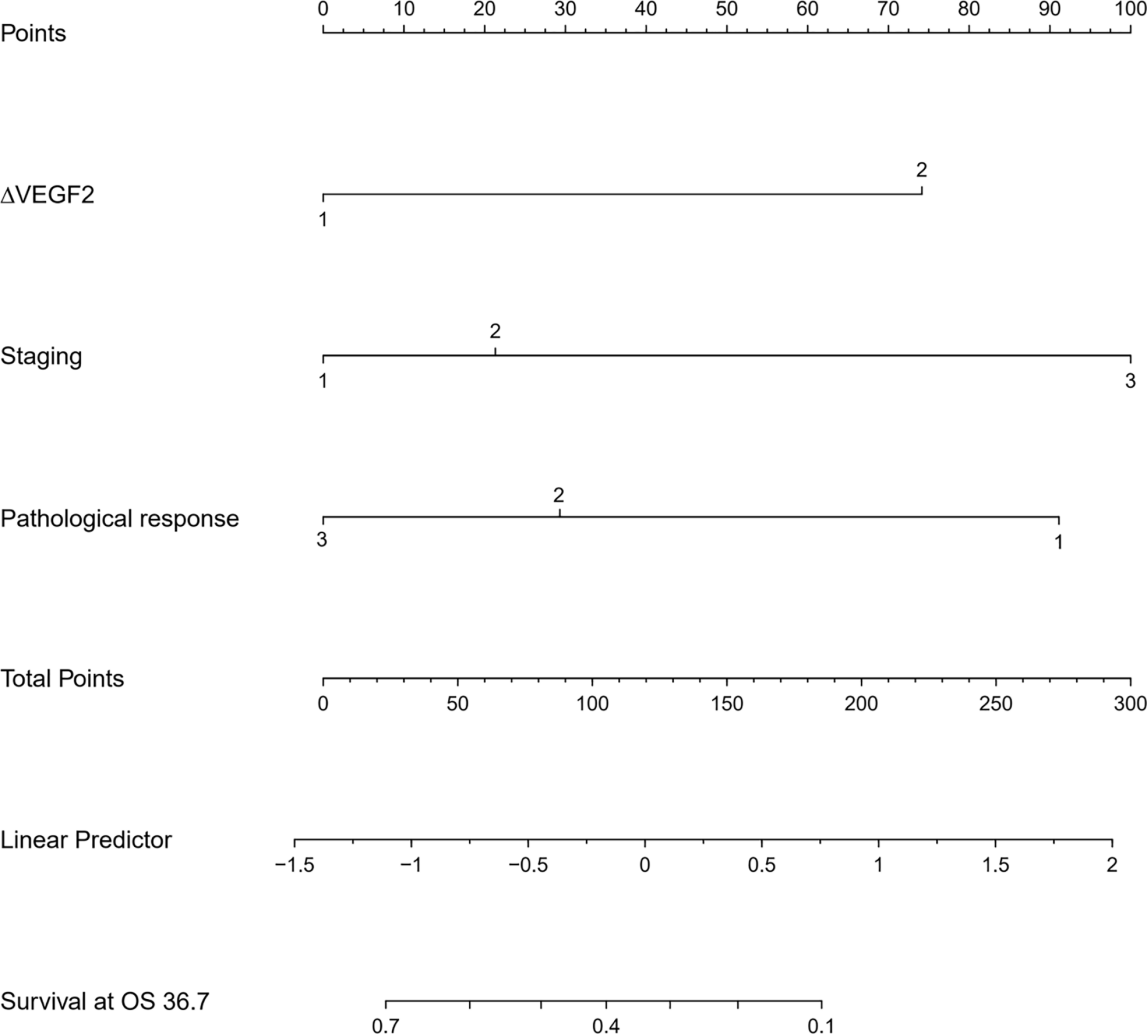
The nomogram for predicting the overall survival of patients with non-surgical ESCC.

**Figure 6:**
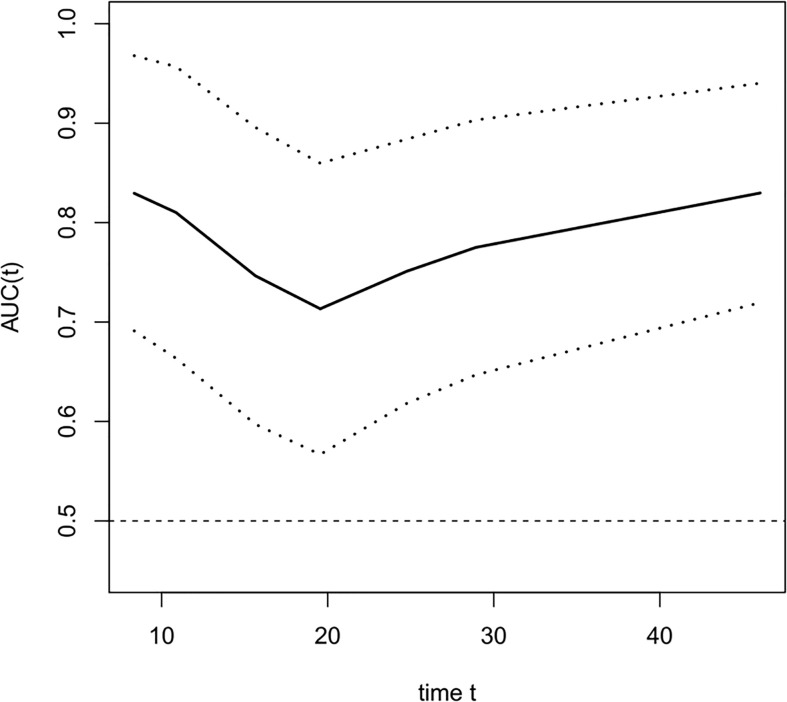
The fitting curve of follow-up time dependent AUC for predicting the overall survival. The solid line is the fitted line, and the dashed lines on both sides are 95% CI. With the extension of follow-up time, AUC was mostly stable, AUC=0.713~0.830.

**Figure 7:**
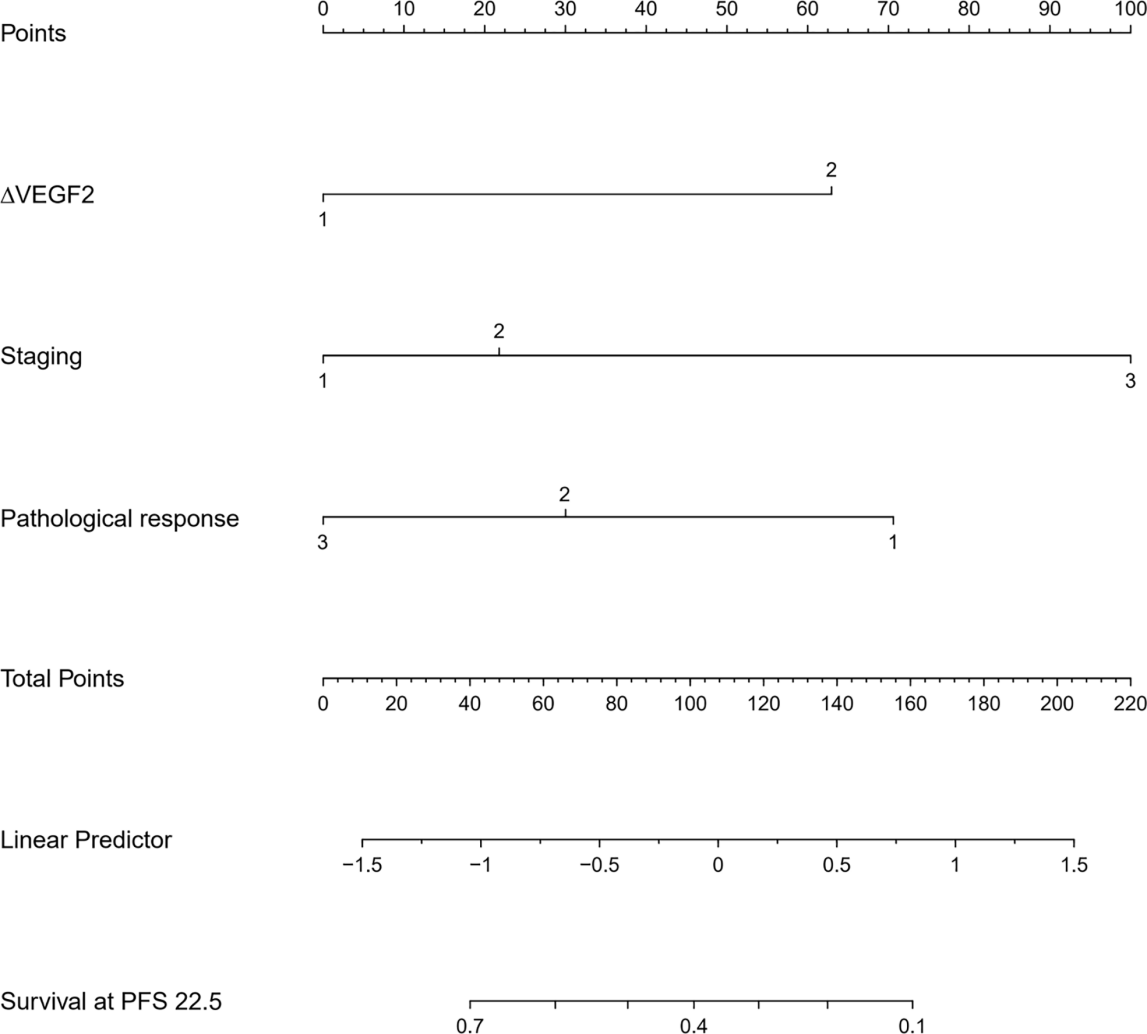
The nomogram for predicting the progression-free survival of patients with non-surgical ESCC.

The COX univariate regression analysis (**Table 5**) showed that, six variables including tumor location, stage, ∆VEGF1, ∆VEGF2, short-term efficacy, and degree of pathological response under gastroscopy were the influencing factors for the OS of non-surgical ESCC patients. The COX multivariate regression analysis showed that, stage, ∆VEGF2, and histopathological response were all independent prognostic factors for overall survival. Patients with stage III, elevated or stable VEGF after treatment, and mild histopathological response had poor treatment efficacy. Based on the above COX multivariate regression analysis, we constructed a prediction model: 0.854 × (∆VEGF2 = 2) + 0.246× (stage=2) + 1.152 × (stage=3)-1.050 × (pathological response=3) -0.712 × (pathological response=2). The model R^2^ was 0.328, and consistency index (C-index) was 0.697. The nomogram (**Figure 5**) and the fitting curve of follow-up time-dependent AUC (**Figure 6**) were plotted. As shown in **Figure 7**, with the extension of follow-up time, the AUC of this model predicting overall survival of non-surgical ESCC patients was mostly stable (AUC=0.713~0.830).

**Table 5:** Univariate and multivariate COX regression analysis of OS in patients with non-surgical ESCC.

Variable	Univariate		Multivariate	
	HR (95% CI)	*P*	HR (95% CI)	*P*
**Tumor location**				
Neck, upper chest (1)	1.0			
Mid chest (2)	1.678 (0.901, 3.126)	0.103	——	——
Lower chest (3)	2.005 (1.030, 3.900)	0.041	——	——
**Staging**				
Stage I (1)	1.0		1.0	
Stage II (2)	1.315 (0.523, 3.305)	0.560	1.661 (0.588, 4.416)	0.354
Stage III (3)	5.745 (2.027, 16.280)	0.001	3.575 (1.126, 11.348)	0.031
**∆VEGF1**				
Reduction group (1)	1.0		——	——
Stable and elevated groups (1)	1.851 (1.131, 3.030)	0.014	——	——
**∆VEGF2**				
Reduction group (1)	1.0		1.0	
Stable and elevated groups (2)	2.094 (1.228, 3.570)	0.007	1.976 (1.110, 3.516)	0.021
**Degree of histopathological response**				
Mild (1)	1.0		1.0	
Moderate (2)	0.316 (0.171, 0.585)	<0.001	0.448 (0.217, 1.055)	0.067
Severe (3)	0.246 (0.125, 0.485)	<0.001	0.322 (0.135, 0.770)	0.011
**Treatment efficacy**				
Non-response (0)	1.0		——	——
Response (1)	0.285 (0.160, 0.508)	<0.001	——	——

**Figure 8:**
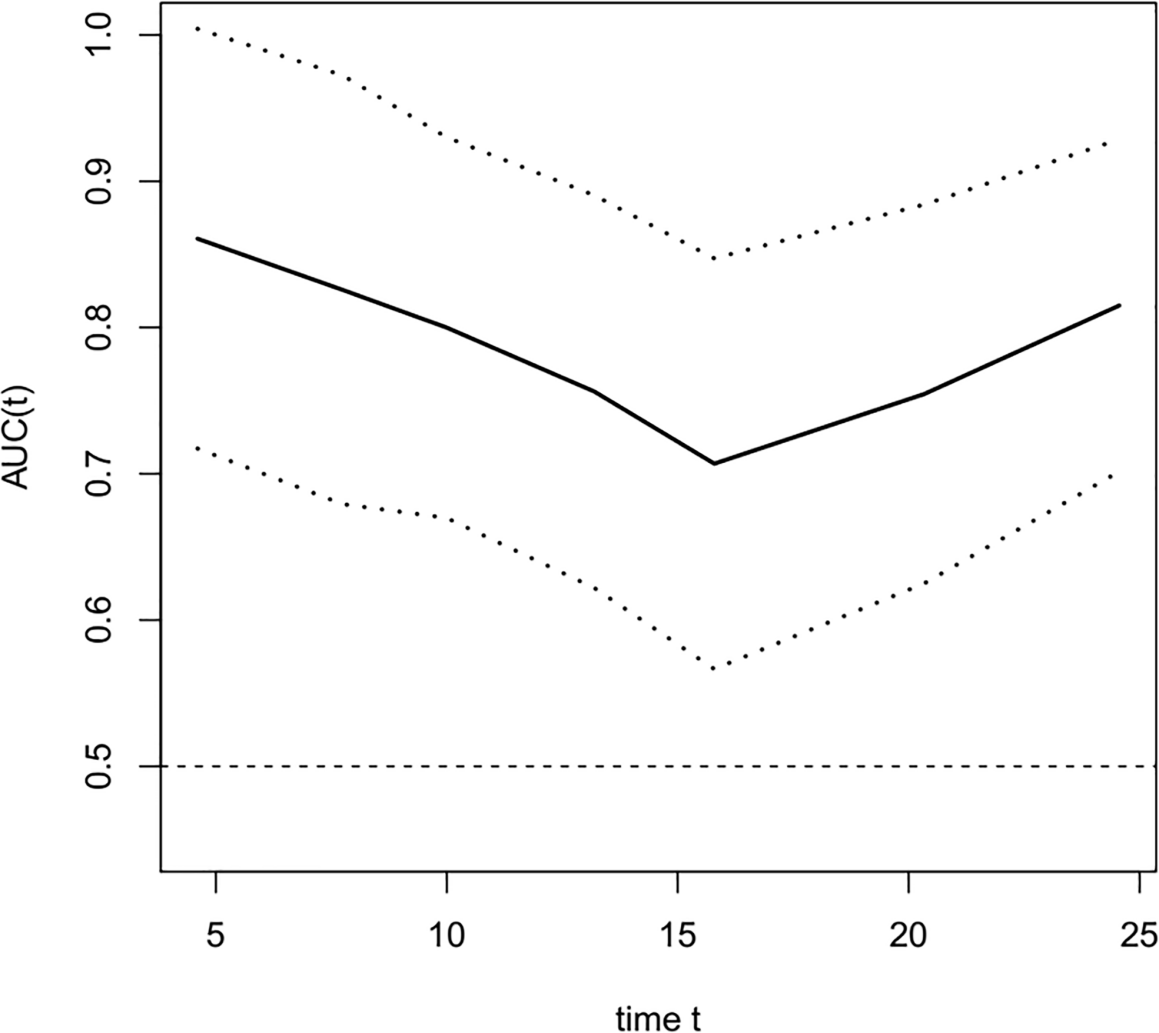
The fitting curve of follow-up time dependent AUC for predicting progression-free survival. The solid line is the fitted line, and the dashed lines on both sides are 95% CI. With the extension of follow-up time, AUC was mostly stable, AUC=0.707~0.861.

The COX univariate regression analysis (**Table 6**) showed that, 6 variables including diabetes, patients’ stage, ∆VEGF1, ∆VEGF2, histopathological response, and short-term efficacy were the influencing factors for progression-free survival of patients with non-surgical esophageal squamous cell carcinoma. The COX multivariate regression analysis showed that, stage, ∆VEGF2, and histopathological response were all independent prognostic factors for patients’ PFS. Patients with stage III, elevated or stable VEGF after treatment, and mild histopathological response had a higher probability of disease progression. Based on the above COX multivariate regression analysis, we constructed a prediction model: 0.974 × (∆VEGF2 = 2) + 0.338× (stage=2) + 1.548 × (stage=3)-1.092 × (pathological response=3)-0.628 × (pathological response=2). The model R^2^ was 0.362, and the C-index was 0.708. The nomogram (**Figure 7**) and a fitting curve of follow-up time-dependent AUC (**Figure 8**) were plotted. As shown in **Figure 8**, with the extension of follow-up time, the AUC of this model predicting the PFS of non-surgical ESCC patients was mostly stable (AUC=0.707~0.861).

**Table 6:** Univariate and multivariate COX regression analysis of PFS in patients with non-surgical ESCC.

Variable	Univariate		Multivariate	
	HR (95% CI)	*P*	HR (95% CI)	*P*
**Diabetes**				
No (0)	1.0		——	——
Yes (1)	2.377 (1.234, 4.580)	0.010	——	——
**Staging**				
Stage I (1)	1.0		1.0	
Stage II (2)	1.345 (0.534, 3.387)	0.529	1.324 (0.515, 3.404)	0.561
Stage III (3)	6.149 (2.166, 17.459)	0.001	4.318 (1.378, 13.534)	0.012
**∆VEGF1**				
Reduction group (1)	1.0		——	——
Stable and elevated groups (2)	1.668 (1.018, 2.733)	0.042	——	——
**∆VEGF2**				
Reduction group (1)	1.0		1.0	
Stable and elevated groups (2)	1.972 (1.153, 3.373)	0.013	2.471 (1.325, 4.610)	0.004
**Degree of histopathological response**				
Mild response (1)	1.0		1.0	
Moderate (2)	0.335 (0.184, 0.613)	<0.001	0.604 (0.284, 1.287)	0.191
Severe (3)	0.236 (0.120, 0.463)	<0.001	0.372 (0.161, 0.858)	0.020
**Treatment efficacy**				
Non-response (0)	1.0		——	——
Response (1)	0.291 (0.165, 0.511)	<0.001	——	——

## Discussion

The radiotherapy-based comprehensive treatment is the main treatment for non-surgical ESCC. However, due to tumor heterogeneity, differences in the study population, sample size, and concurrent chemotherapy regimens, there are large differences in ORR after treatment (53.3%~98.3%) between different clinical studies (6). Although the ORR of the enrolled patients in this study reached 75.0% after treatment, how to screen out cases that can respond to a specific treatment is one of the problems faced by clinicians. The serum biomarkers have the advantages of simplicity, non-invasiveness, and dynamic detection. In recent years, studies on ESCC serum biomarkers have identified a variety of cancer-related molecules, including autoimmune antibodies against tumor-associated antigens (TAAs), microRNAs, non-coding RNAs, cytokines (interferons, interleukins, growth factors, etc.), circulating tumor cells, and circulating tumor DNA (20). Different ESCC serum biomarkers indicate different disease states and tumor information, exhibiting different advantages and disadvantages in clinical applications.

Serum VEGF belongs to the platelet-derived growth factor family and is overexpressed in 31%-60% of esophageal patients (15), and its overexpression is usually associated with late disease stage or poor prognosis (21). However, there are still controversies regarding the evaluation and prediction of short-term efficacy using serum VEGF for non-surgical ESCC. Yoon et al. (21) found that ESCC patients with low VEGF expression before anti-tumor therapy had a high probability of incomplete response after treatment. Lu et al. (22) retrospectively analyzed a small sample set and found that the short-term efficacy in ESCC patients was not significantly correlated with the level of VEGF before treatment, and the ESCC patients whose serum VEGF decreased after treatment showed good short-term efficacy. In this study, we found that the serum VEGF level during radiotherapy is the independent factor for predicting short-term efficacy in ESCC patients: low serum VEGF level during radiotherapy indicates good short-term efficacy. The mechanism may be related to the decreased tumor burden and invasiveness after treatment resulting in reduced secretion of VEGF from tumor to the peripheral circulation (15, 23). Although serum VEGF to predict short-term treatment efficacy in ESCC patients had high sensitivity (93.7%), the specificity was low (42.9%), suggesting that it alone is unreliable in predicting short-term efficacy. Therefore, in clinical practice, the serum VEGF should be combined with clinical characteristics of ESCC patients (such as TNM stage, treatment methods, etc.) to judge the efficacy comprehensively.

Prognostic biomarkers are related to prognosis, death, or other clinical outcomes. They are often used to help determine which patients are suitable for a particular type of treatment. So far, there is no clinically evidence-based biomarker for predicting the prognosis of esophageal cancer (24), and the relationship between serum VEGF and prognosis in ESCC patients is still controversial (25). Shimada et al. reviewed 82 ESCC patients and found that the 3-year survival rate of patients with high serum VEGF levels (greater than 451 pg/mL) before treatment was significantly lower than that of patients with low VEGF levels (less than 451 pg/mL); the 3-year survival rates in those two groups were 13% and 54%, respectively (15). Some studies have (20, 26) found that there was no significant difference in survival between low and high VEGF expression groups before antitumor therapy, and they proposed that serum VEGF was not a prognostic marker for predicting ESCC related death. However, Wang et al. (16) and Chen et al. (27) found that VEGF kinetics was the prognostic factor for locally advanced ESCC patients receiving curative CCRT. In this study, we found that the change in serum VEGF levels after radiotherapy (∆VEGF2) was an independent influencing factor for OS and PFS in ESCC patients, which was similar to the study of Wang et al. (16) and Chen et al. (27). Patients with significantly reduced serum VEGF after radiotherapy showed a better prognosis. More importantly, we constructed a new prognostic model for non-surgical ESCC based on ∆VEGF2. In addition, this study also found that the patients with stage III and mild histopathological response showed poor prognosis.

Digestive endoscopy is a commonly used examination method in evaluating ESCC treatment. Studies have found that (16, 28, 29), whether the ESCC patients can pass the endoscopy after treatment, and the degree of endoscopic pathological response is closely related to the prognosis; the more severe the pathological response, the better the prognosis. This study also found that the degree of endoscopic pathological response was an independent influencing factor for the OS and PFS of non-surgical ESCC patients. Since the esophagus is a hollow organ, there is a potential risk of gastrointestinal bleeding and esophageal perforation during endoscopic lesion biopsy. We confirmed that none of the patients had gastrointestinal bleeding or perforation in this study.

Nomogram is the integration of multiple predictive indicators, which can be used to diagnose or predict disease onset, progression and prognosis. Since nomogram is intuitive and highly operable, it has been widely used to predict tumor treatment efficacy and prognosis (25, 30, 31). Hou et al. (25) found that the expression level of VEGF was a predictor for distant metastasis, OS and metastasis-free survival in patients with ESCC; the COX regression model constructed in conjunction with VEGF expression, tumor stage and cell grading could predict the risk of metastasis after surgery. Although this model was validated in the validation group, they did not provide the model equation or indicators for model evaluation. Tang et al. (31) combined age, gender, pathological type, degree of differentiation, number of metastases, and treatment methods to construct a model for predicting cancer-related survival in metastatic esophageal cancer, with a C-index of 0.762. In this study, we constructed a model for predicting OS and PFS in patients with non-surgical ESCC by combining the patients’ stage, change of serum VEGF after radiotherapy (∆VEGF2), and the degree of endoscopic histopathological response during treatment. The C-Index of our model was 0.697 and 0708, respectively. By plotting the fitting curve of follow-up time-dependent AUC, we found that, with the extension of follow-up time, the prediction power of this model was stable, and the AUC was greater than 0.7.

This study still has the following limitations. First, this study was a single-center study with a small sample size, and it was impossible to isolate a trial validation group for internal and external validation. Second, although it has been confirmed that serum VEGF levels are in good agreement with tissue VEGF expression, it would be more accurate to use ESCC tissue VEGF expression to construct the model. Third, since the esophagus is a hollow organ, endoscopic lesion biopsy during treatment can potentially cause gastrointestinal bleeding and esophageal perforation, which should be used with caution.

## Conclusion

For patients with non-surgical ESCC, those with low VEGF levels during radiotherapy or significant VEGF reduction after radiotherapy had better treatment efficacy. Moreover, it is feasible to construct a model to evaluate and predict the efficacy and prognosis of non-surgical ESCC patients based on serum VEGF measurement. Therefore, the dynamic measurement of serum VEGF levels in patients with non-surgical ESCC is beneficial.

## Data availability statement

The original contributions presented in the study are included in the article/supplementary material. Further inquiries can be directed to the corresponding authors.

## Ethics statement

The studies involving human participants were reviewed and approved by the Affiliated Changzhou No. 2 People’s Hospital of Nanjing Medical University. The patients/participants provided their written informed consent to participate in this study.

## Author contributions

LH and JY contributed to conception and design of the study. QM conducted data collection and patients follow-up. LH and FS carried out data statistics and analysis. ZK and MZ wrote the manuscript. JY revised the manuscript. All authors read and approved the final manuscript.

## Funding

The study was supported by Major Science and Technology Project of Changzhou Municipal Health Commission (ZD202017); Changzhou Sci&Tech Program (No. CZ20210030).

## Conflict of interest

The authors declare that the research was conducted in the absence of any commercial or financial relationships that could be construed as a potential conflict of interest.

## Publisher’s note

All claims expressed in this article are solely those of the authors and do not necessarily represent those of their affiliated organizations, or those of the publisher, the editors and the reviewers. Any product that may be evaluated in this article, or claim that may be made by its manufacturer, is not guaranteed or endorsed by the publisher.
